# Clinical Notes on Galvanism

**Published:** 1888-11

**Authors:** C. M. Ramsdell

**Affiliations:** Lampasas, Texas


### CLINICAL NOTES ON GALVANISM.

By C. M. Ramsdell, A. M., M. D., Lampasas, Texas.

[Read in Section of Electro-Therapeutics at the Galveston Meeting of the Texas State Medical Association. Published by authority of the writer.]

THE CASE BOOK of the country doctor cannot be expected to furnish material for an elaborate article on electro-therapeutics, nor does the writer wish, even were he able to do so, to theorize upon the workings of electricity in the various forms in which it is used in medicine; but he submits the following report of cases, in the hope that the record may prove serviceable to some of those who read it.

CASE I.—Mrs. Mary S., a farmer's wife, aet 30, a healthy looking woman, mother of five children. She complained of weakness in the back with severe pains in the stomach, worse during the day-time; the latter symptom being her principal trouble. Her former physicians had given her various drugs for the stomach trouble, but withont effect. Careful questioning and examinations failing to discover anything wrong with the digestive organs except the one symptom of pain, I turned my attention elsewhere and soon found marked tenderness on pressure over the last three dorsal and first lumbar vertebræ. She then told me that she had a fall from her horse some months before, which had laid her up for a few days with a lame back, and that her stomach had never troubled her before that time. Rest in bed was enjoined, a moderately strong current from a Kidder Tip

Battery used daily, half an hour at a time, for twelve days. At the end of this time the pains in the stomach had disappeared, and the tenderness in the spine was so far relieved that the patient was allowed to leave her bed for a part of each day, but the daily use of the battery was continued a week longer and its applications every two or three days for a month. A perfect recovery took place without a single dose of any kind of drug. In this case the positive electrode was placed upon the spine over the affected part and the negative over the epigastrium.

CASE II.—Miss Jennie H., a dressmaker, aged 27, of large frame, but spare, and in poor health generally. This young lady was subject to severe head-aches, apparently of nervous origin. A mild, continuous current directed latterally from temple to temple and, sometimes, from the forehead to the nape of the neck, nearly always gave complete relief in fifteen or twenty minutes. When used in the last named direction an electrode ending in a flat, metallic disc was placed on the back of the neck. and the forehead stroked with a large sponge-electrode.

CASE III.—Judy S., colored, a nurse, aged 40, the mother of seven children. Two years after her last child was born—four months previous to consulting me—she noticed a difficulty in menstruation, the flow seeming to be retarded, and she suffered pain, a thing before unknown to her on such occasions. Each monthly period after that became the occasion of increasing pain until at the last it became so intense as to cause her to seek medical aid. Examination disclosed a tumor which I diagnosed as an intra-uterine fibroid, apparently as large as a small orange, which seemed to act like a ball-valve, occluding the neck of the uterus. Its point of attachment I did not discover. Without any well-defined idea of what I expected to accomplish, I subjected her daily and sometimes twice a day to strong, interrupted currents from the tip battery, one electrode, the negative, being passed up the uterine canal till it touched the tumor, the other being applied to the spine in the lumbar regions. At first but little effect was seen, but after a few sittings strong contractions began to be felt as soon as the current was used, and on the eleventh day, the os having been quite patulous when the electrode was introduced, the contractions forced the fibroid out into the vagina, where it remained hanging by a slender pedicle. Improvising an ecrasseur with a loop of slender brass wire which I had bought to snare fish with, and a piece of brass tubing, I soon removed the tumor. The patient had no more dysmenorrhœa.

CASE IV.—J. A. R., a cattle raiser, aged 57. This gentleman, the writer's father, had suffered with a lame back for several years, the result of repeated falls from horses and of over work. Crossing the Colorado during a rise, his horse fell with him, wetting him completely. He rode eight miles before reaching shelter, a norther blowing, and the result was that he was confined to his bed during a large part of the winter. When able to be on his feet again he engaged in hard work, such as felling trees, digging wells, etc., against medical advice, with the result that the next winter he took his bed, not to leave it again for nearly three years. His symptoms were severe pains in the lumbar region and along the sciatic nerves; persistent wakefulness, even when the pain was relieved by opiates, and intense nervous excitement, particularly in the lower extremeties. During the first winter little medicine was given, the pain being relieved by immersion in hot water, a bathtub having been provided in which the patient's whole body could be immersed. After several months, a tumor was discovered over the last lumbar and first sacral vertebra, which, from its appearance having been preceded by a rigor, and from slight fluctuations being noticeable, was diagnosed as an abscess from caries of the spine. Galvanism was then used for several months, a moderately strong induced current being the most grateful to the patient, affording complete relief from pain during its continuance, and for an hour or two afterwards. A favorite way of using the battery was to apply the positive electrode to the nape of the neck, and the negative to the sole of the foot, changing from one foot to the other. Under the use of galvanism the tumor over the spine disappeared, and the nervous excitement of the lower extremeties was greatly diminished. The patient was also enabled to sleep better than before. The use of the battery was discontinued during the last year of his illness, it seeming to have largely lost its effect; but it is my opinion that the absorption of the abscess and the arrest of the destructive process in the spine was wholly due to its use. He has now for several years enjoyed a fair degree of health.

CASE V.—William B., aged 35, an Irish, railroad section hand. He had been on a drunk for several days, and wound up by taking six grains of morphine at one dose. I found him unconscious, half an hour after taking the morphine, his pupils contracted to the size of a pinhead, respiration 7 per minute, pulse 150, very feeble. He could not be made to swallow anything. A very strong, interrupted current from a Kidder galvanic battery was passed through his arms and legs, the pain soon rousing him into a semi-conscious state, from which he immediately relapsed into a profound stupor whenever the current was withdrawn. The battery was used, at short intervals, for five or six hours, when the patient was so far recovered as to be able to dispense with it.

CASE VI.—Norah Harper, aged 20, a stranger in this city, having quarreled with her husband, she swallowed about seven fluid drachms of laudanum, and was not found until an hour afterward. Her pupils were then almost obliterated, her respirations four per minute; pulse could not be counted. A very powerful current was applied by touching both electrodes to the skin close together, producing an intense burning sensation. They were applied every few seconds for just an instant, on her face, hands and arms, breast and lower extremities. Lead pencils were also placed between her fingers and hard pressure made by squeezing her fingers together. This was discontinued after she began to give signs of feeling the pain, and the electricity continued through the night, about ten hours in all. She recovered so as to be able to ride out with her husband in the afternoon of that day.

In sciatica, in rheumatism, in many forms of neuralgia, I find nothing so good for relieving the pain as the galvanic current. In fact, I believe, that the practitioner, who has once learned the use of a good battery, would as soon think of doing without a hypodermic syringe or fever thermometer, as it.

It beats pills for relieving constipation ; it soothes pain as effectually as, and far more safely and pleasantly as to after-effects, than opium ; it will, in many cases, produce sleep as surely as chloral ; and it excels phosphorus, strychnia and all that class of drugs in restoring disordered nerves to a normal condition. As a discutient, absorbent and resolvent it has no equal. If electricity has not been proven to be identical with nerve force, it at least has so much similarity to it that the physician, who well understands its use, has at his command a remedial agent, before whose powers all other single means of affecting the human organism, fade into insignificance. It produces pain, it relieves pain ; it causes muscular contractions, it produces relaxations ; it gives sleep, it insures wakefulness in opposition to the effect of the strongest hypnotics; it is a most powerful nerve stimulant, it soothes and calms the most violent nervous excitement. While other therapeutic means have come into use, flourished, waned and disappeared, electricity has steadily gained ground ; its sphere of application is continually widening, and its many excellencies are becoming better known.

